# Supplementary material for: Tail proteins of phage SU10 reorganize into the nozzle for genome delivery
Source: Nat Commun. 2022 Sep 24;13:5622. doi: 10.1038/s41467-022-33305-w (PMC9509320; doi:10.1038/s41467-022-33305-w)

Supplementary material for:

Tail proteins of phage SU10 reorganize into the nozzle for genome delivery

Marta Šiborová[1], Tibor Füzik[1], Michaela Procházková[1], Jiří Nováček[1], Martin Benešík[2], Anders S. Nilsson[3], Pavel Plevka[1]

[1] Central European Institute of Technology, Kamenice 753/5, 625 00 Brno, Czech republic
[2] Faculty of Science, Masaryk University, Kamenice 753/5, 625 00 Brno, Czech republic
[3] Department of Molecular Biosciences, The Wenner-Gren Institute, Stockholm University, 106 91 Stockholm, Sweden

Content:

Supplementary figures
Supplementary tables
Cover images for supplementary movies

Supplementary figures:

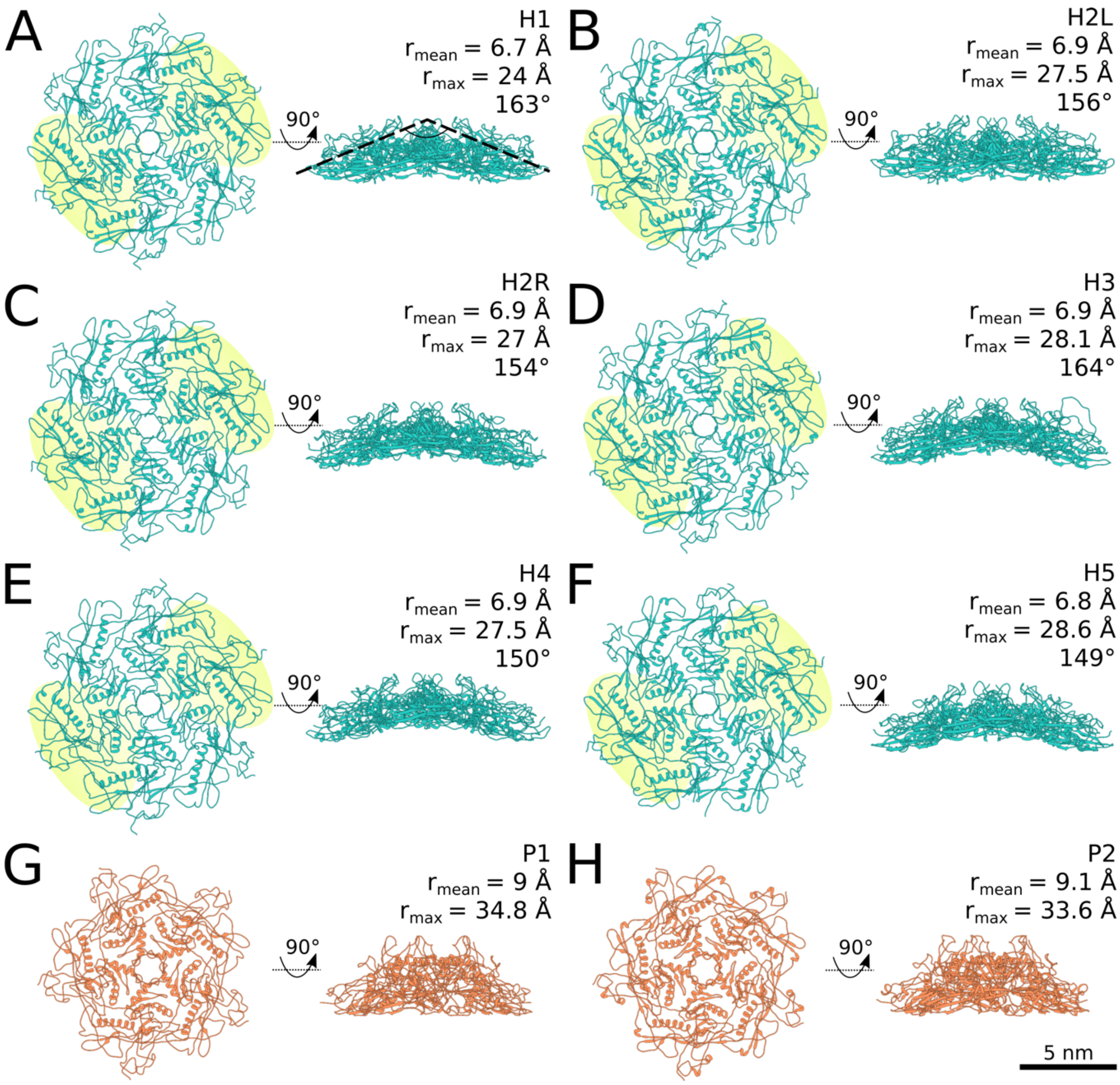

Supplementary Fig. 1. Comparison of different types of major capsid protein hexamers and pentamers in SU10 capsid.

(A-F) Hexamers (turquoise) and **(G, H)** pentamers (orange) of major capsid proteins are shown in cartoon representation. Hexamer and pentamer types, based on their position in the prolate capsid, are labeled as in Fig. 3. Each panel shows the top and side view of the oligomer. The planarity of each of the assemblies was characterised by fitting it with a least squares plane and calculating the mean (r_{mean}) and maximal residual distance (r_{max}) of atoms forming the structure to the plane. The curvature of hexamers was further characterized by fitting least squares planes (highlighted in lime green) to pairs of major capsid proteins located on opposite sides of a hexamer and calculating the angle between the planes. The pairs of hexamers selected for least squares fitting were selected to show the maximum curvature of each hexamer. The two planes of each hexamer are only shown in the top views. The dashed lines in the side view of panel A are shown to indicate the angle between the least squares planes.

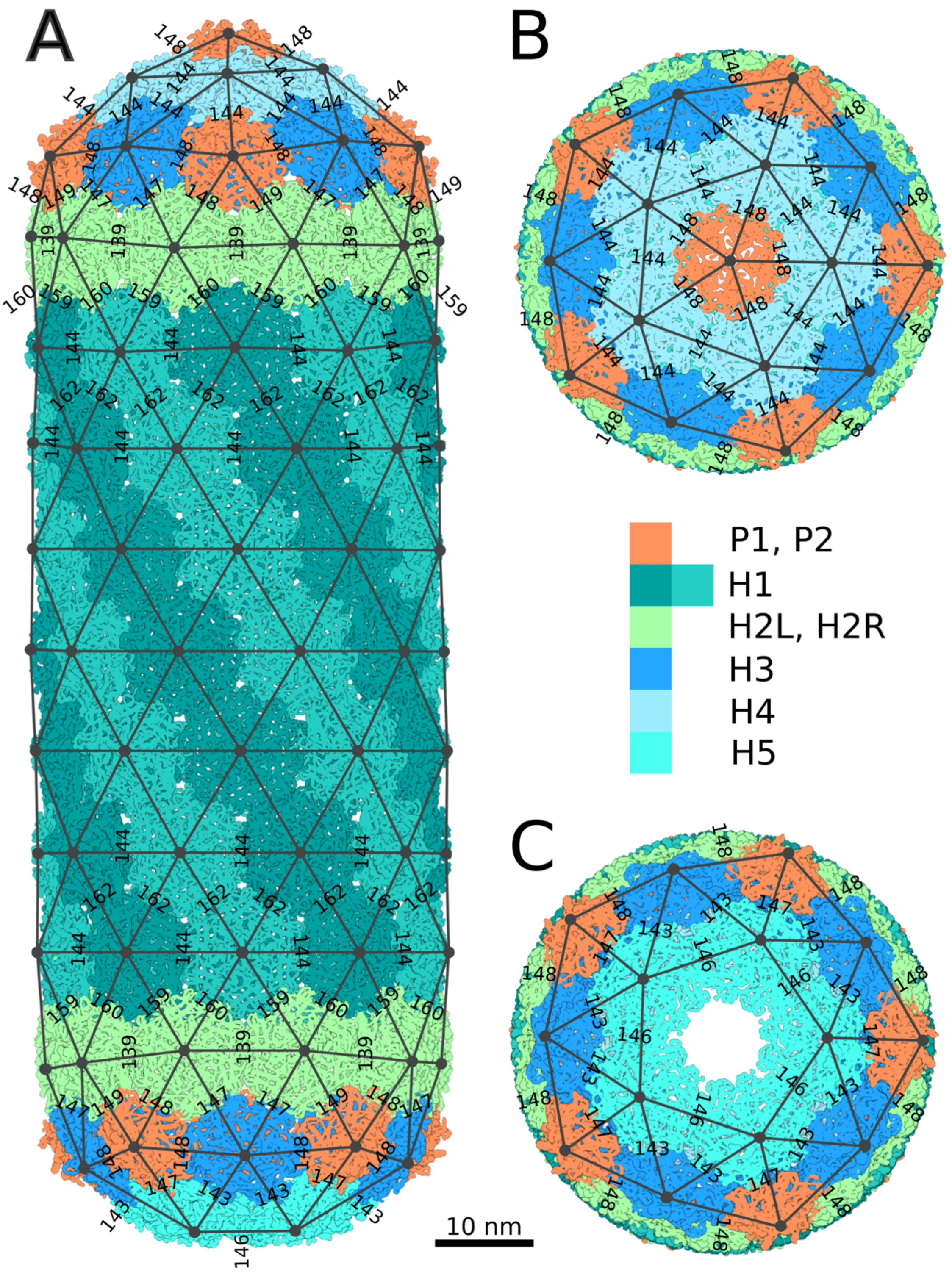

Supplementary Fig. 2. Angles between hexamers and pentamers in SU10 capsid.

SU10 capsid viewed perpendicular to its long axis **(A)**, capsid cap without tail **(B)**, and capsid cap with tail **(C)**. In panel C, the tail proteins and portal were omitted to enable a clear view of capsid proteins. Individual types of hexamers and pentamers are color coded as indicated in the legend. Centers of hexamers and pentamers, indicated with black dots, are connected by black lines to emphasize the capsid's organization. Numbers printed across the lines indicate the angles between the least squares planes of hexamers or pentamers connected by that particular line.

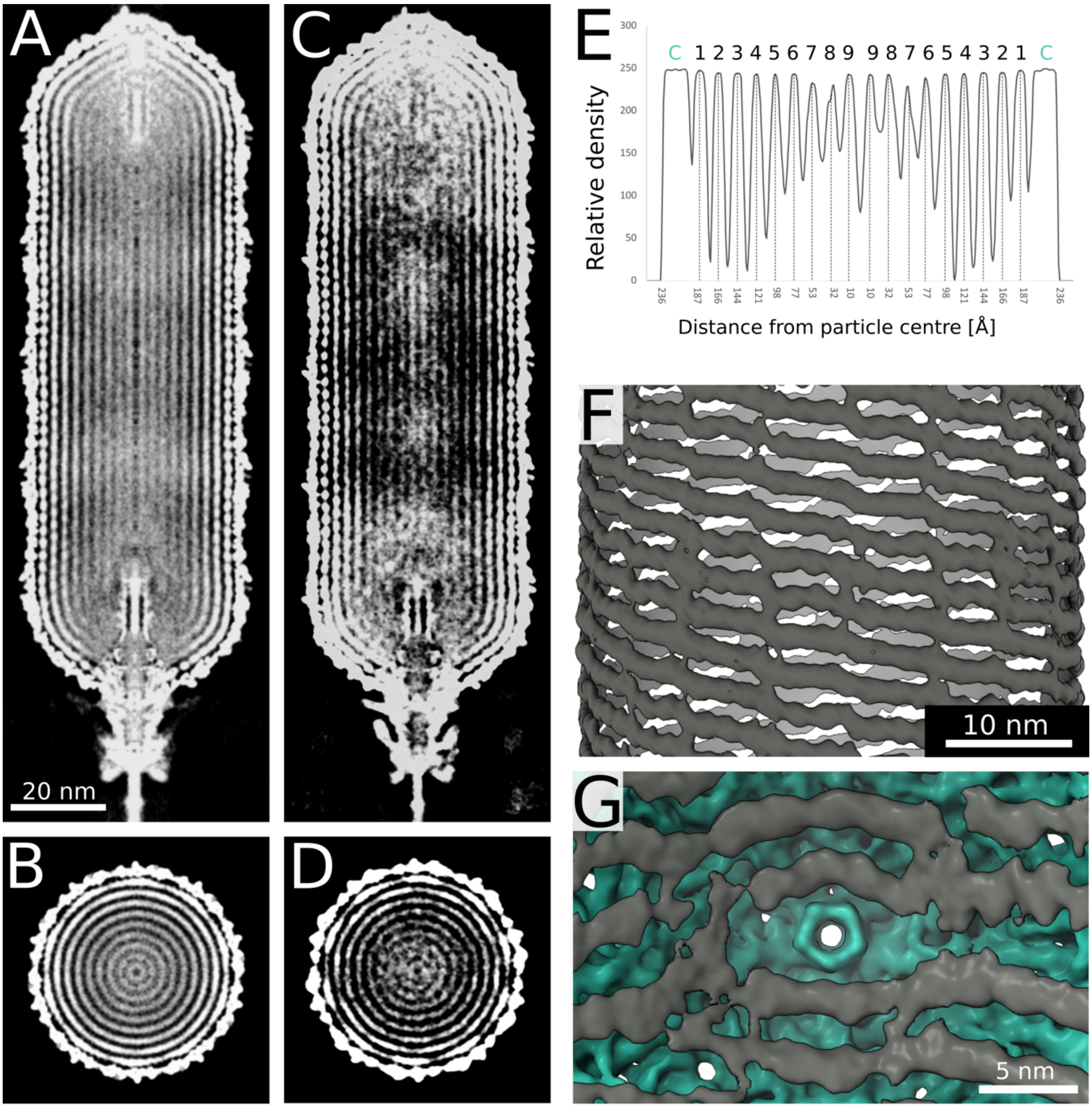

Supplementary Fig. 3. Packaging of dsDNA genome in SU10 virion.

(AB) Central sections from cryo-EM reconstruction of SU10 virion with imposed fivefold symmetry along (A) and perpendicular (B) to long axis of particle. **(CD)** The same as AB, but from asymmetric reconstruction of SU10 virion. **(E)** Profile of average cryo-EM density values from virion reconstructed with imposed fivefold symmetry plotted along line crossing capsid centre. The density of the capsid is indicated with a turquoise C. The virion contains nine layers of density corresponding to the genome and inner core proteins. **(F)** The outermost genome density layer is resolved into strands corresponding to dsDNA. The map is shown at 5σ. **(G)** The DNA strands from the outermost genome layer bypass the protrusion formed by a P2 pentamer. The view is along the pseudo-fivefold axis of the P2 pentamer from inside the particle. The density of capsid proteins is shown as a turquoise surface, the DNA density as a grey surface. The maps of capsid and DNA are shown at 1.8σ and 2.5σ, respectively.

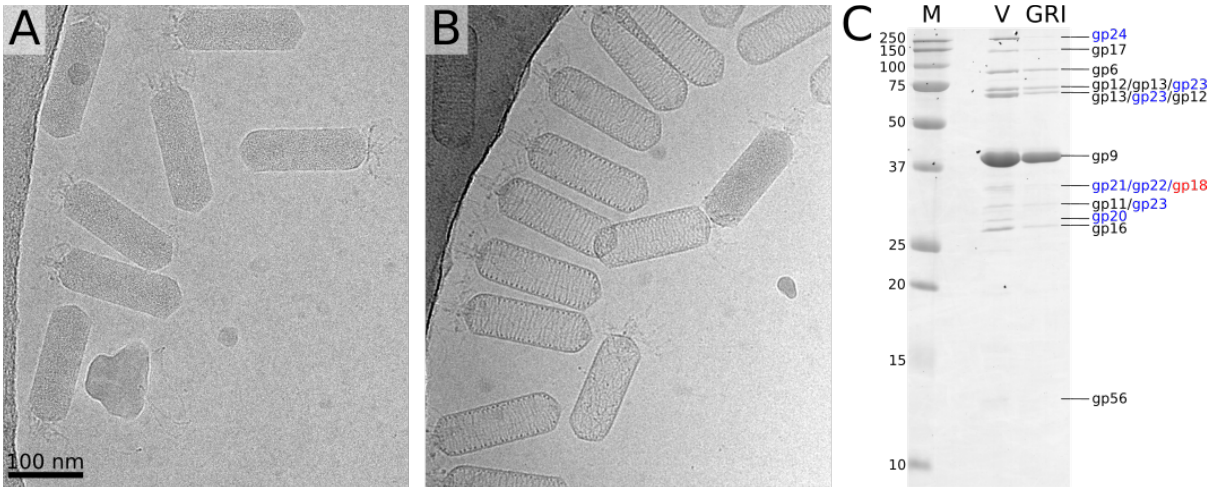

Supplementary Fig. 4. **Induction of SU10 genome release *in vitro.***

(A) Electron micrograph of virions of bacteriophage SU10. **(B)** Particles of phage SU10 induced to release their genomes *in vitro*. DNA remaining in the genome release intermediates interacts with the capsid. In addition, a few DNA strands are stretched between the particle poles. Electron micrographs of SU10 virions and genome release intermediates were recorded from three and two independent particle preparations, respectively. **(C)** SDS PAGE of proteins from virions (V) and genome release intermediates (GRI) of bacteriophage SU10. The protein composition of individual bands was determined using mass spectrometry, and is indicated by gene product numbers. Genome release intermediates lack putative core proteins (gp20-24; blue) and tail needle protein (gp18; red). Numbers to the left of the marker (M) line indicate molecular weights in kDa. SDS PAGE gels of SU10 virions and genome release intermediates were repeated twice using independent particle preparations.

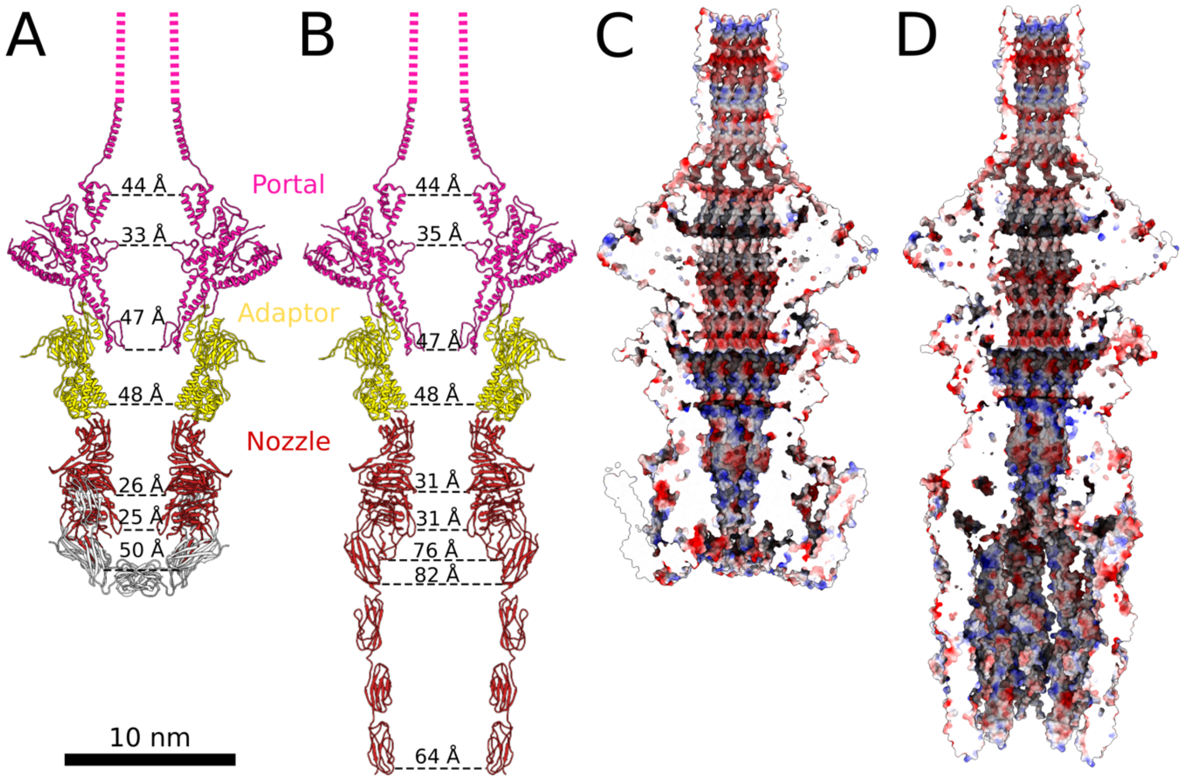

Supplementary Fig. 5. Properties of tail channel of SU10 virion and genome release intermediate.

(A, B) Cartoon representations of proteins forming tail channel in SU10 virion (A) and genome release intermediate (B). Parts of nozzle proteins extending to the front and back of the tail in the SU10 virion are shown in white. In the virion, the narrowest constrictions are formed by the channel valve loops of the portal proteins and loops of the beta-propeller domains of nozzle proteins. In the genome release intermediate, the beta-propeller domains of nozzle proteins are shifted away from the tail axis, resulting in the tail channel broadening. **(C, D)** Molecular surface representation of tail proteins of (C) SU10 virion and (D) genome release intermediate colored according to the electrostatic potential (scale +−10 kcal / (mol e^{-})). The front halves of the complexes were removed to show the electrostatic potential of the tail channel. The clipped volumes of protein subunits are shown in white.

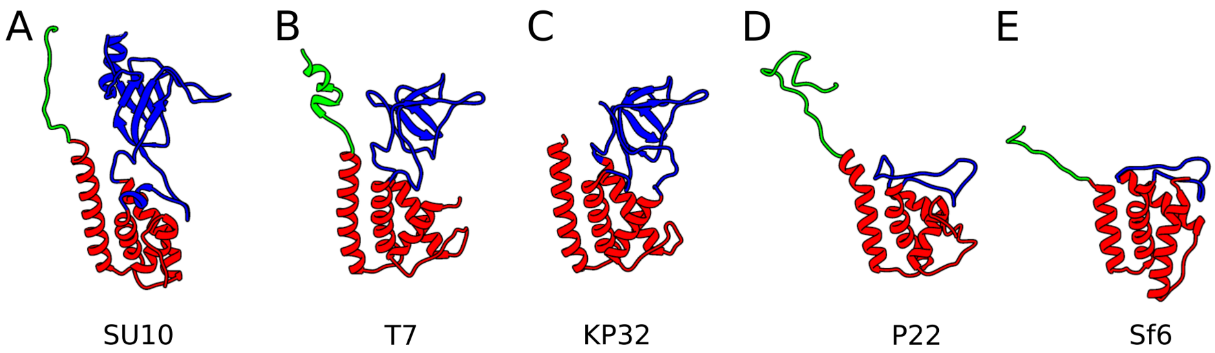

Supplementary Fig. 6. Comparison of structures of adaptor proteins of tailed phages.

Cartoon representations of structures of adaptor proteins of bacteriophages **(A)** SU10, **(B)** T7 (PDB: 6R21), **(C)** KP32 (PDB: 5MU4), **(D)** P22 (PDB: 4V4K), and **(E)** Sf6 (PDB: 5VGT). The proteins are colored according to the domain composition: the embracing loop is shown in green, helix bundle in red, and long tail fiber dock in blue.

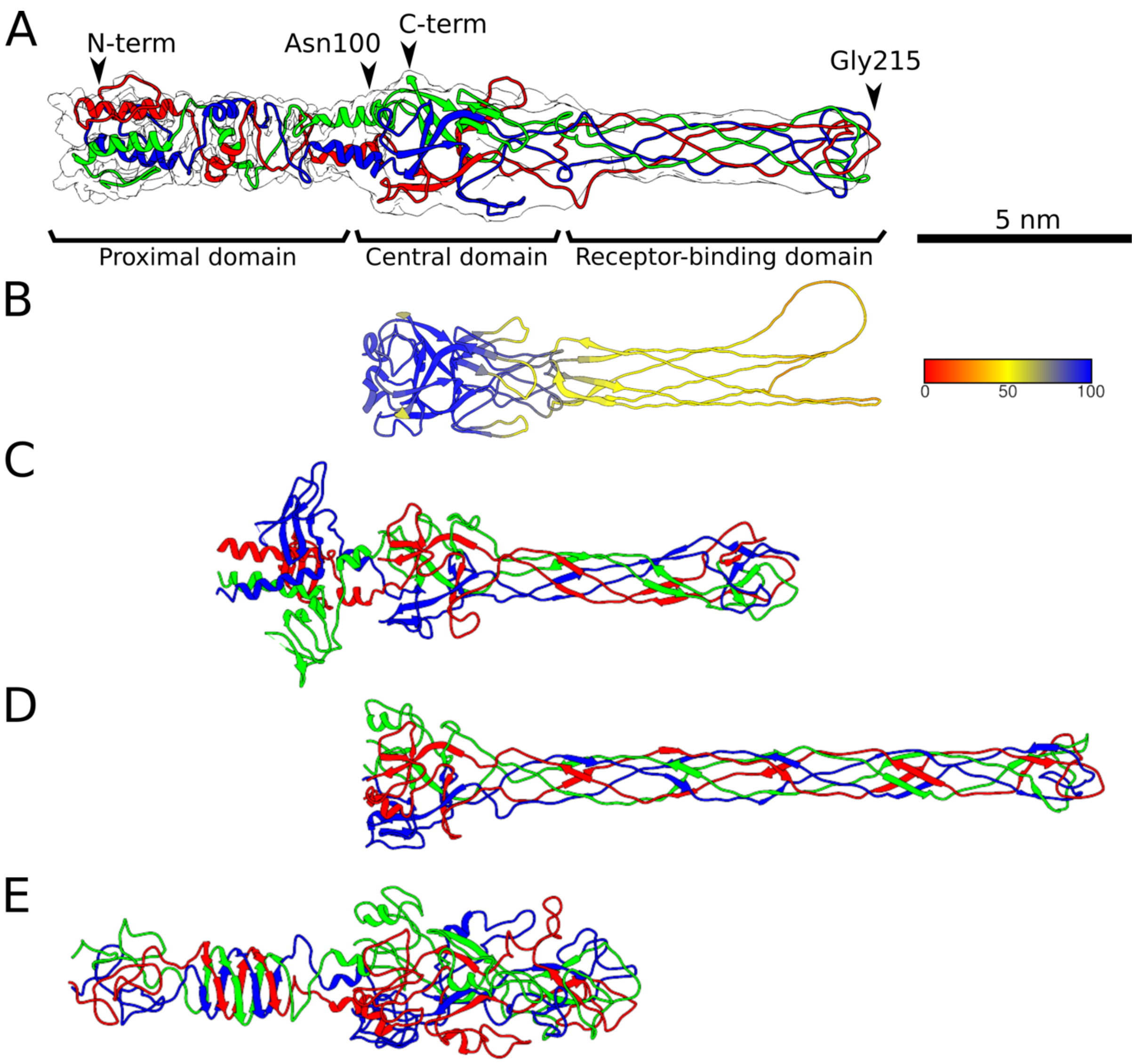

Supplementary Fig. 7. Comparison of structures of tail fibers of tailed phages.

(A) Cartoon representation of short tail fiber of bacteriophage SU10 fitted into cryo-EM density reconstruction. The proximal domain was built into the cryo-EM map, whereas the central and receptor-binding domains were modeled using AlfaFold2 multimer. The subunits forming the trimer structures of the fibers are shown in red, blue and green. The division of the short tail fiber into domains is indicated. The proximal, central, and distal parts of short tail fiber density are shown at contour levels 4σ, 2σ, and 1σ, respectively. Central and receptor-binding domains fit into the corresponding densities with correlation coefficients of 0.69 and 0.55, respectively. **(B)** Central and receptor-binding domains of SU10 short tail fiber modeled using AlfaFold2 multimer, colored according to pLDDT scores (high values indicate reliable structure prediction). **(C)** Receptor-binding protein of temperate bacteriophage JUB59 (PDB: 6OV6). **(D)** Receptor-binding tip of long tail fiber (gp10) of phage T4 (PDB: 2XGF). **(E)** C-terminal domain of short tail fiber (gp12) of bacteriophage T4 (PDB: 1PDI).

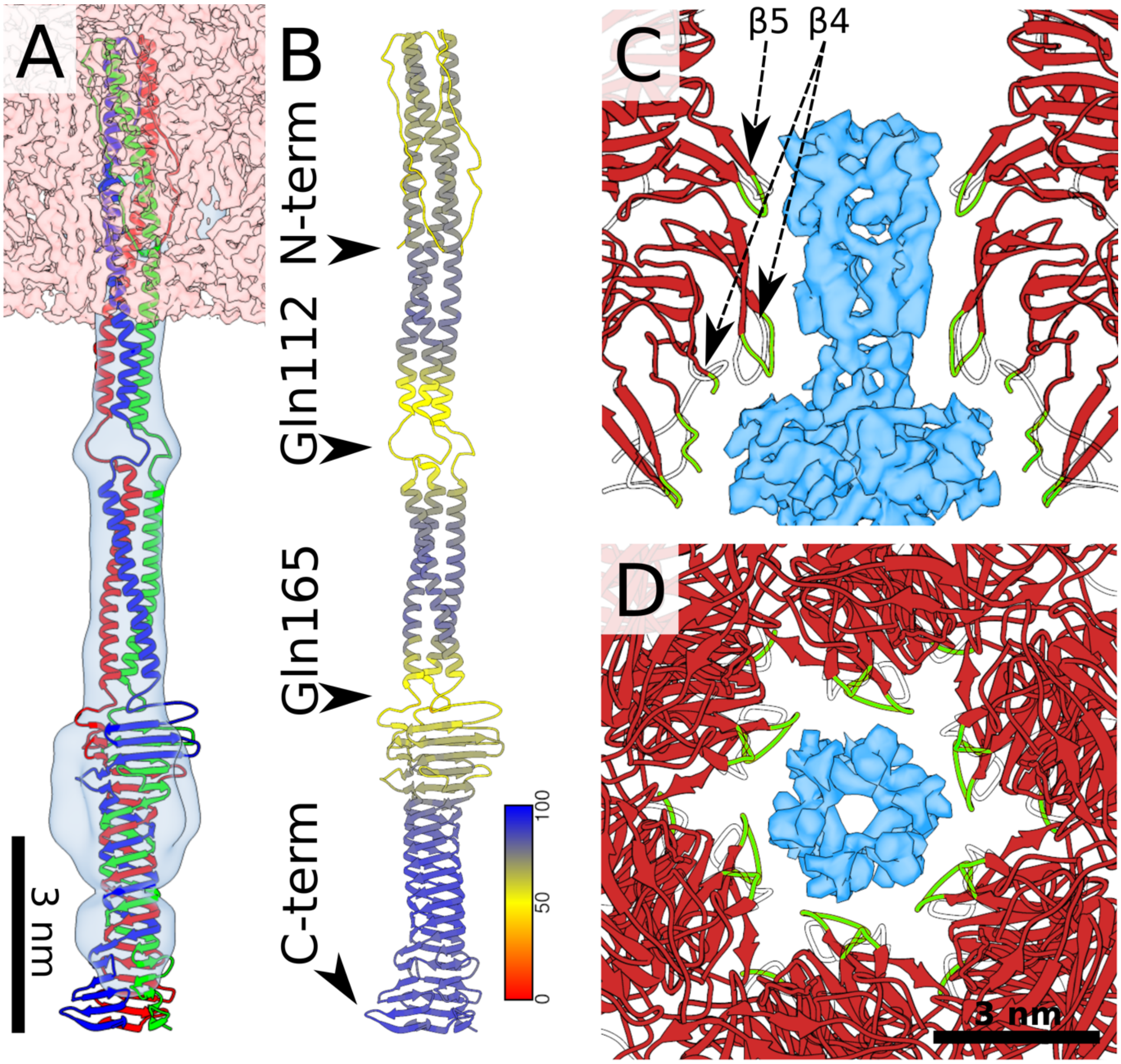

Supplementary Fig. 8. Structure of SU10 tail needle.

(A) Cartoon representation of SU10 tail needle modeled using AlfaFold2 multimer fitted into corresponding part of cryo-EM map of SU10 virion. The predicted structure was split into two fragments (residue range 1-164 and 165-322) that were fitted independently, resulting in a correlation coefficient between the model and map of 0.81. The cryo-EM map of the tail needle is shown as a semi-transparent blue surface at 1σ. The density of nozzle proteins, which interact with the tail needle, is shown at a contour level of 4.5σ as a semi-transparent red surface. **(B)** Cartoon representation of tail needle modeled using AlfaFold2 multimer, colored according to pLDDT scores (high values indicate reliable structure prediction). **(C, D)** Interactions of nozzle proteins shown in cartoon representation with tail needle shown as blue density surface at contour level of 3σ. The beta-propeller domains of nozzle proteins are shown in red. Loops of nozzle proteins that mediate the contacts with the tail needle in SU10 virion are shown in green. The structure of the superimposed nozzle proteins from the genome release intermediate, lacking the tail needle, is shown in white. View perpendicular to the tail axis (C) and along the tail axis from outside the virion (D).

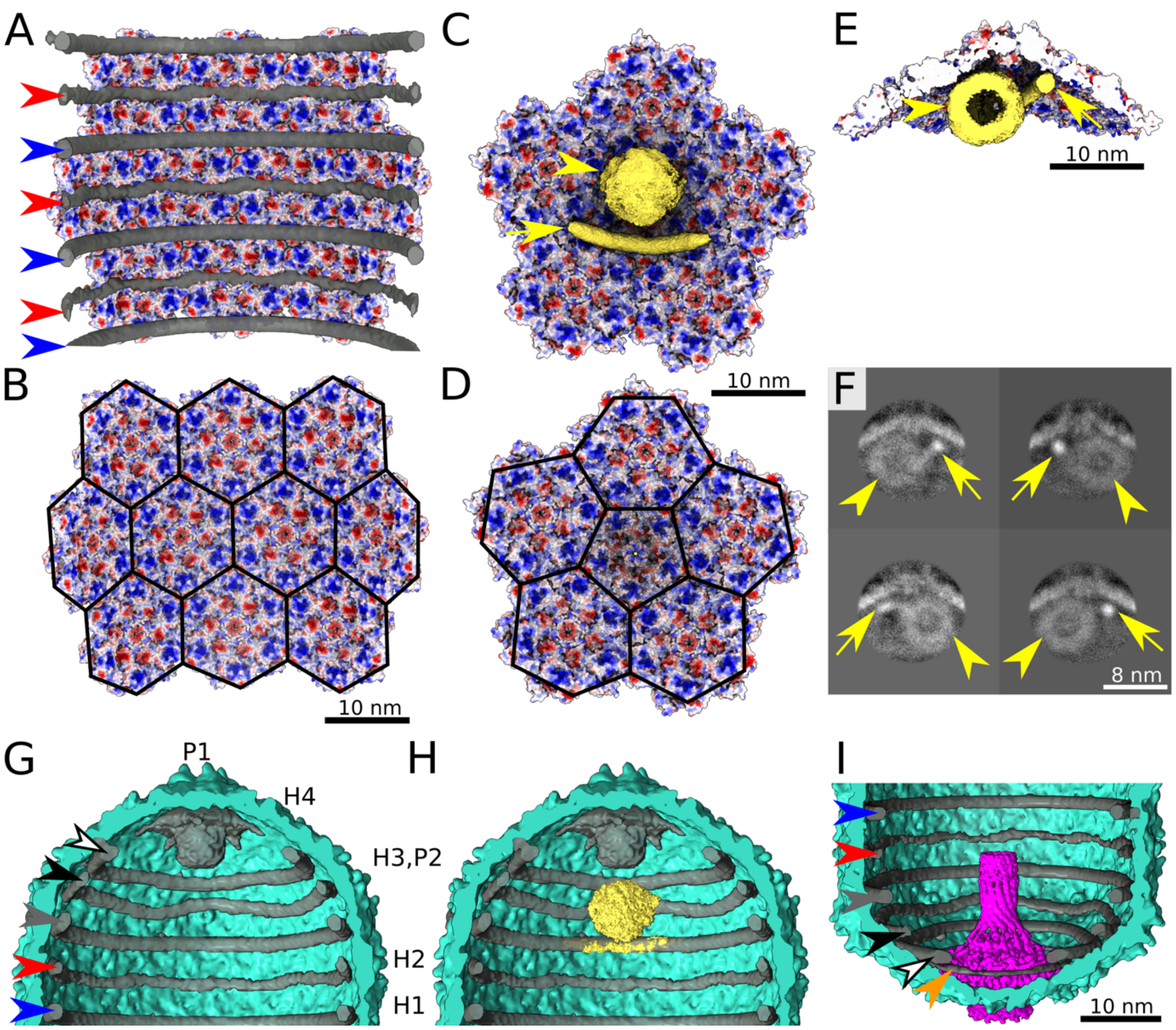

Supplementary Fig. 9. Organization of DNA remaining in genome release intermediate of SU10.

(A, B) Detail of DNA strands interacting with positively charged surface of tubular part of SU10 capsid. Capsid proteins are shown in surface representation colored according to electrostatic potential +−10 kcal/(mol e$^-$), and the DNA density is shown as a grey surface. There are two types of DNA densities that interact with each H1 hexamer of capsid proteins. For the numbering of pentamers and hexamers in the SU10 capsid, see Fig 3. The first type interacts with the centers of hexamers, has a wiggly structure, and is indicated with a red arrowhead. The second type of DNA density interacts with the edges of hexamers, is straight, has a stronger density than the first type, and is indicated with a blue arrowhead. In B, the DNA density was omitted, and borders of the hexamers are highlighted with a black chicken wire net. **(C, D)** Segment of DNA, indicated with yellow arrow, and hollow sphere of density with diameter of 80 Å, indicated with yellow arrowhead, interact asymmetrically with inner surface of P1 pentamer. The map of the sphere is shown at 3σ. In D, the DNA and sphere densities were omitted, and the borders of the P1 pentamers and H4 hexamers are highlighted with a black chicken wire net. **(E)** Side view of structure shown in panel C with front half removed. **(F)** 2D class averages from asymmetric reconstruction of capsid cap that does not contain a tail. Yellow arrows indicate the DNA density segment, and arrowheads the hollow spheres of electron densities. **(G)** Arrangement of DNA density in SU10 genome release intermediate associated with capsid cap opposite the tail. The density of the capsid is shown as a turquoise surface, and that of DNA as a grey surface. The front part of the reconstructions has been removed to show the capsid interior. The two types of DNA strands associated with the H1 hexamers are indicated with a red and blue arrowhead as in panel A. Three additional strands with distinct structures are associated with the cap. The third type of DNA strand is straight and interacts with the border between P2 pentamers and H2L and H2R hexamers, as well as the border between H3 hexamers and H2L

and H2R hexamers, and is indicated with a grey arrowhead. The fourth type of DNA strand interacts with P2 pentamers and H3 hexamers, twists around the beta-barrels formed by the annular loops of capsid proteins forming the pentamers, and is indicated with a black arrowhead. The fifth type of DNA strand binds to the positively charged border between the P1 pentamer and H4 hexamers, and is indicated with a white arrowhead. The maps of capsid and DNA are shown at 8σ and 30σ, respectively. **(H)** Superposition of putative DNA density (yellow) associated with P1 pentamer of capsid proteins with P2 pentamer indicates that DNA segment interacting with the P1 pentamer binds to positively charged surface at border between P1 pentamer and H4 hexamer analogous to the third type of DNA strand, described in panel G. The map of the capsid, sphere, and DNA are shown at 8σ, 3σ, and 8σ, respectively. **(I)** Arrangement of DNA density in SU10 genome release intermediate associated with portal complex. The density of the capsid is shown as a turquoise surface, portal in magenta, and DNA in grey. The front part of the reconstructions has been removed to show the capsid interior. The arrangement of genome density associated with the portal is similar to the genome density in the capsid cap opposite the tail. Additionally, one extra ring of DNA density, indicated with an orange arrowhead, interacts with the portal complex via its wing domains. The map of capsid, portal, and DNA are shown at 8σ, 2σ, and 8σ, respectively.

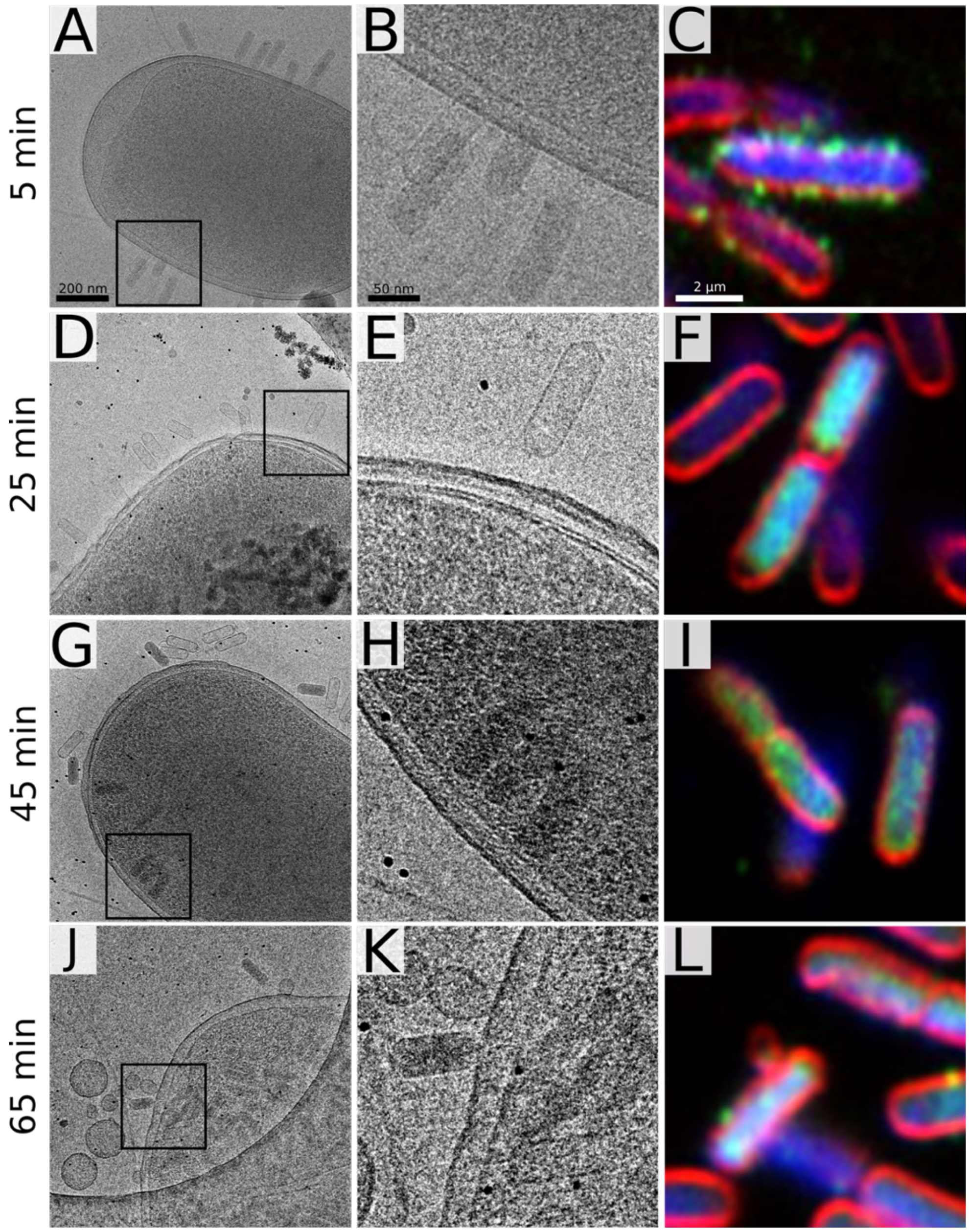

Supplementary Fig. 10. Infection cycle of phage SU10 visualized *in situ* using cryo-ET and fluorescence microscopy.

(A, D, G, J) Cryo-electron tomograms of SU10-infected *E. coli* cells at various times post-infection. Black squares indicate positions of details shown at higher magnification in the central column of the figure **(B, E, H, K). (C, F, I, L)** Confocal fluorescence microscopy images of SU10-infected *E. coli* cells. The *E. coli* genome was labeled using DAPI (blue), *E. coli* membranes were stained using SynaptoRed (red), and DNA packed in SU10 particles was stained with DmaO (green). **(A, B, C)** Attachment of SU10 virions to *E. coli* cells 5 minutes post-infection. **(D, E, F)** Phage DNA has been delivered into host cells, and empty particles remain attached at the cell surface 25 minutes post-infection. **(G, H, I)** Assembly of progeny virions in infected cells 45 minutes post-infection. **(J, K, L)** Cells filled with newly assembled phage virions 65 minutes post-infection. At 65 minutes post-infection, both tomograms and fluorescence images show membrane vesicles that may have been shed from the infected cells. More than fifteen infected cells were visualized using cryo-ET for each observation time. The fluorescence microscopy was performed in two biological replicates and more than 10 acquisition areas were visualized for each observation time.

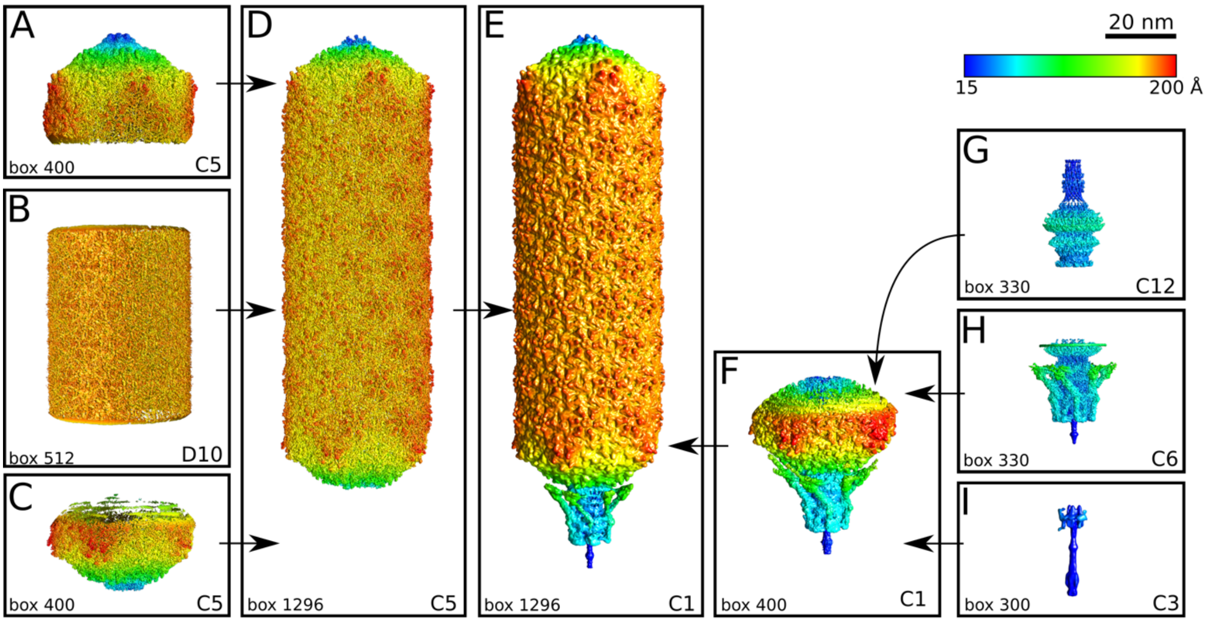

Supplementary Fig. 11. Combination of sub-particle reconstructions for assembly of composite SU10 virion structure.

Symmetries and box sizes are indicated for each reconstruction. **(A, B, C)** Sub-particle reconstructions of segments of SU10 capsid. **(D)** Reconstruction of complete capsid with five-fold symmetry. **(E)** Asymmetric reconstruction of SU10 virion. **(F)** Asymmetric reconstruction of capsid-tail interface. **(G, H, I)** Sub-particle reconstructions of SU10 portal, tail, and tail needle.

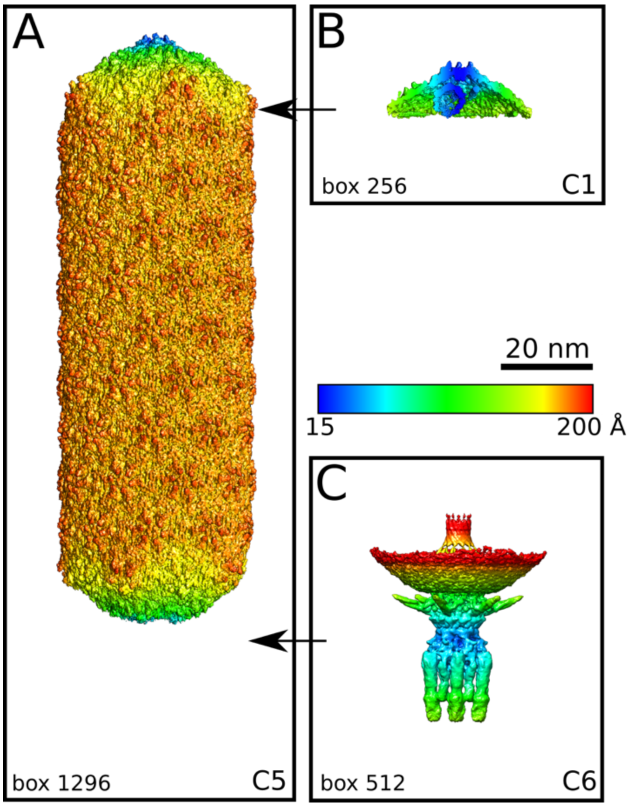

Supplementary Fig. 12. Combination of sub-particle reconstructions for assembly of composite structure of SU10 genome release intermediate.

Symmetries and box sizes are indicated for each reconstruction. **(A)** Reconstruction of complete capsid with fivefold symmetry. **(B)** Asymmetric reconstruction capsid cap. **(C)** Subparticle reconstruction of tail.

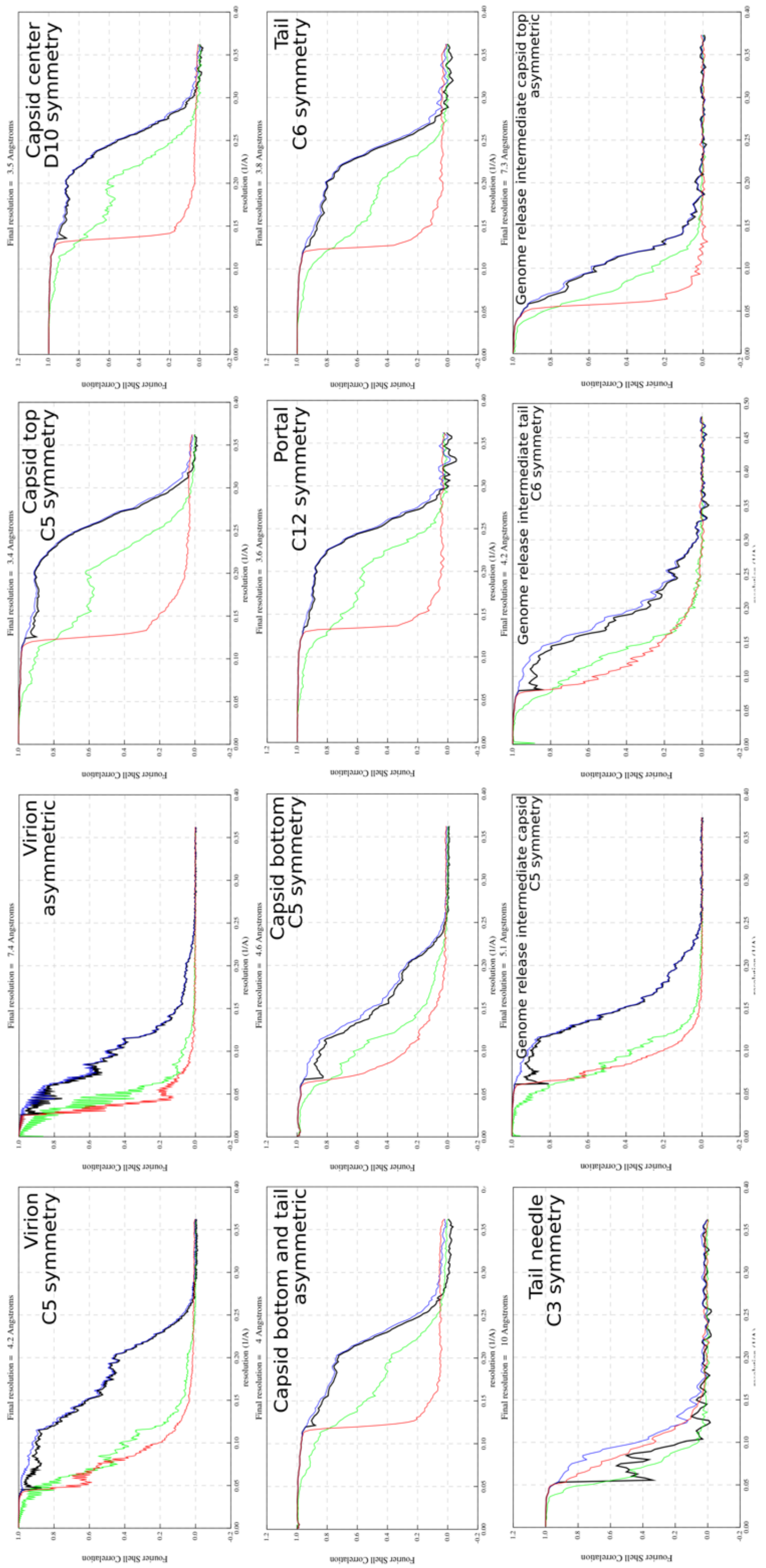

Supplementary Fig. 13. FSC curves of cryo-EM reconstructions.

Each panel shows a comparison of Fourier shell correlation curves of Fourier shell correlation corrected half-maps (black), unmasked half-maps (green), masked half-maps (blue), and phase randomized and masked half-maps (red).

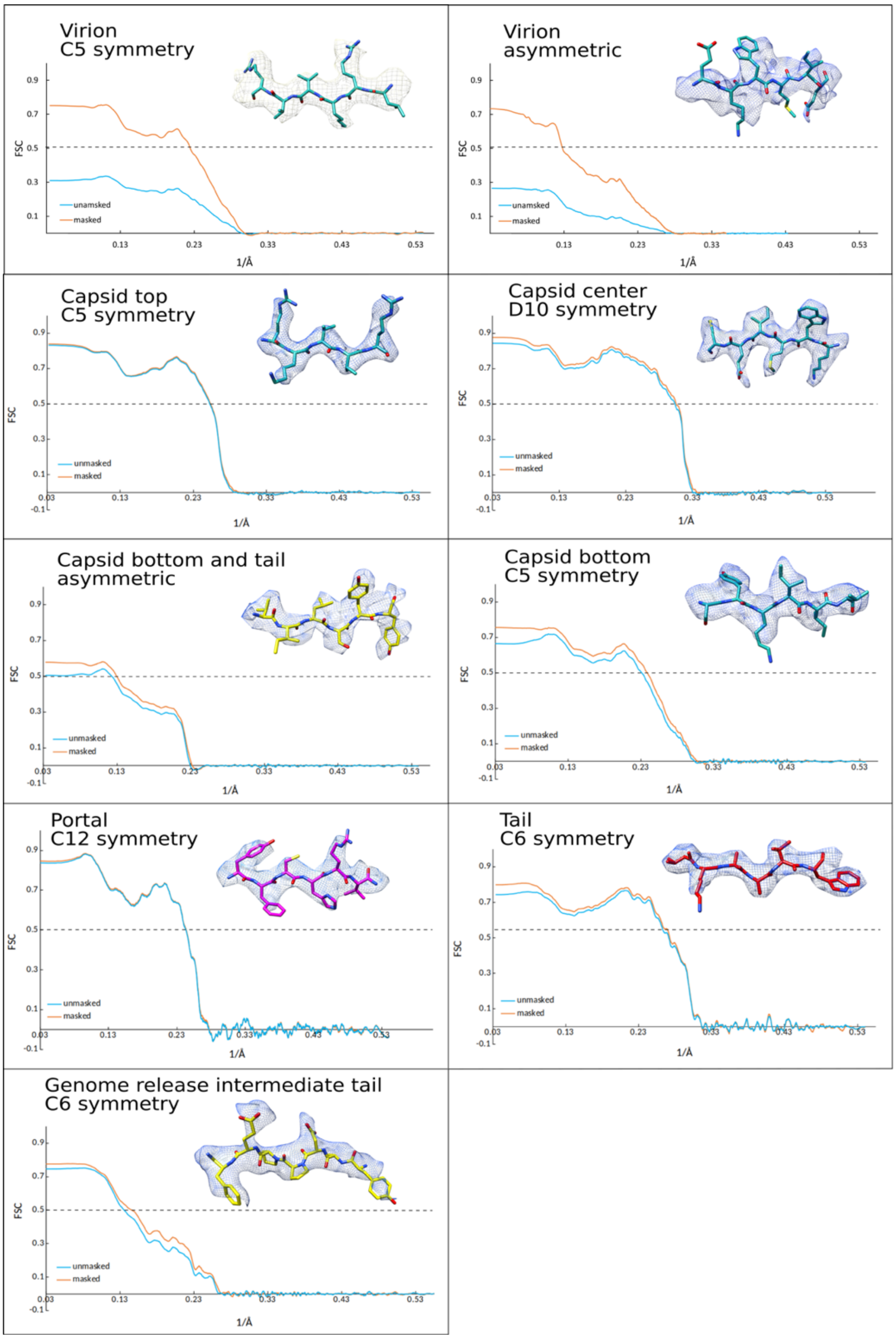

Supplementary Fig. 14. Plots of the map to model FSC and examples of model fit to Cryo-EM densities.

The values of the map to model FSC were calculated using Phenix - comprehensive validation with an automated masking option enabled. For the map of virion reconstructed with C5 symmetry, the model included asymmetric parts of capsid top, capsid center, and capsid bottom. For the asymmetric map of the virion, the model included asymmetric parts of the capsid top, capsid center, capsid bottom, and tail. For the remaining maps, the models were expanded according to their symmetry.

Supplementary tables

ORF	Protein	In virion	Detected by MS	Number of residues	Sym.	Count in virion	Number of modeled residues	Built residues	Model completeness	PDB codes of homologus proteins
1	Hypothetical Protein	no	no	76						
2	Hypothetical Protein	no	no	106						
3	Hypothetical Protein	no	no	276						
4	Hypothetical Protein	no	no	201						
5	Terminase Large Subunit	no	no	513						
6	**Portal**	yes	yes	747	C12	12	604	6-144, 189-246, 256-662	81	all phage portals
7	Hypothetical Protein	no	no	78						
8	Scafolding Protein	no	no	358						
9	**Major Capsid Protein**	yes	yes	352	C5, oblate	715	347 (hexon)<br>338 (penton)	3-349<br>11-348	98.6<br>96	all HK97 folds
10	Ig-like Domain	no	no	132						1CWV
11	**Adaptor**	yes	yes	250	C6 dodecamer	12	227<br>229	3-105, 113-238<br>3-105, 113-236	90.8<br>91.6	6R21, 5MU4, 3J4B
12	**Long Tail Fibers**	yes	yes	786	six trimers	18	86<br>79<br>67	3-88<br>12-91<br>24-91	11<br>10<br>8.5	1PDI, 1OCY, 1H6W
13	**LongTail Fiber distal part**	yes	yes	723	six trimers	18	0	0	0	3GW6, 6F45, 6EU4, 3GUD
14	Holin	no	no	76						
15	Lysin	no	no	163						
16	**Short Tail Fibers**	yes	yes	267	six trimers	18	267<br>(partialy by AplphaFold2)	2-100<br>101-267	100	2FKK, 5HX2, 2XGF,<br>1OCY, 1PDI, 5IV5
17	**Nozzle Protein**	yes	yes	1005	C6	6	912	2-375<br>452-648<br>664-1005	90.7	2FTX, 1UIC
18	**Tail Needle**	yes	yes	322	C3	3	322<br>(AlphaFold2)	1-322	100	4LIN
19	Hypothetical Protein	no	no	343						
20	**Transglycosylase**	yes	yes	260						4CFP, 4P0G, 5O29, 5A5X
21	**Hypothetical Protein**	yes	yes	350						
22	**DNA Injection Protein**	yes	yes	322						
23	**Internal Virion Protein**	yes	yes	515						
24	**Nuclear Pore Complex**	yes	yes	1473						

Supplementary Table 1. List of structural proteins of phage SU10.
Horizontal thick lines in the table indicate borders of polycistronic mRNAs.

	capsid c5	capsid c1	capsid top	capsid center	capsid bottom	capsid bottom and tail c1	neck c12	tail c6	tail needle	composite map
**Data collection and processing**										
Detector	Falcon3	Falcon3	Falcon3	Falcon3	Falcon3	Falcon3	Falcon3	Falcon3	Falcon3	Falcon3
Magnification	59000	59000	59000	59000	59000	59000	59000	59000	59000	59000
kV	300	300	300	300	300	300	300	300	300	300
Exposure [e-/A2]	49	49	49	49	49	49	49	49	49	49
Pixel size	1.38	1.38	1.38	1.38	1.38	1.38	1.38	1.38	1.38	1.38
Symmetry	C5	C5	C5	D10	C5	C1	C12	C6	C3	C1
Initial number of particles	25689	25689	25689	25689	25689	25689	25600	25600	19111	
Final number of particles	9418	9418	22146	25689	25648	11688	17296	19111	9564	
Initial model	Averaged tomogram	C5 map of native virion low-pass filtered to 30A	de novo	de novo	de novo	C12 map of native neck low-pass filtered to 30A	de novo	de novo	de novo	
Map resolution	4.2	7.4	3.4	3.5	4	4.6	3.6	3.8	10	
FSC treshold	0.143	0.143	0.143	0.143	0.143	0.143	0.143	0.143	0.143	
**Database entry**										
EMDB	EMD-14488	EMD-14492	EMD-14485	EMD-14484	EMD-14487	EMD-14489	EMD-14483	EMD-14486	EMD-14909	EMD-14977
PDB	7Z49	7Z4B	7Z46	7Z45	7Z48	7Z4A	7Z44	7Z47		
**Refinement**										
Atoms (except hydrogens)	254288	284810	159496	31962	45140	65650	9458	29092		
Residues	32560	37451	10367	2077	5831	8414	598	1885		
B-factor	56.4	64.79	89.97	32.37	51.62	61.4	136.17	48.71		
FSC model 0.5 (masked)	4.55	8.13	4.07	3.35	4.35	7.92	6.86	3.87		
**RMSD**										
Bond length [A]	0.004	0.009	0.002	0.004	0.002	0.004	0.002	0.003		
Bond angles [°]	0.908	1.141	0.503	0.893	0.534	0.913	0.386	0.54		
**Validation**										
MolProbity score	1.99	2.08	1.95	1.76	1.88	2.07	1.46	1.88		
ClashScore	8.38	10.16	6.29	4.63	6.09	10.56	4.33	5.84		
C-beta outliers [%]	0	0.4	0	0	0	0.01	0	0.06		
Poor rotamers [%]	0.48	0.87	0	1.23	0	0	0.77	0		
**Ramachandran plot**										
Outliers [%]	0.05	0.35	0.07	0	0.05	0.05	0	0.05		
Favoured [%]	90.44	89.74	88.01	92.69	90.1	90.71	96.26	89.56		

Supplementary Table 2. **Cryo-EM structure quality indicators of SU10 virion**

	capsid c5	tail c6	capsid top	composite map
**Data collection and processing**				
Detector	K2	K2	K2	K2
Magnification	105000	105000	105000	105000
kV	300	300	300	300
Exposure [e-/A2]	52	52	52	52
Pixel size	1.34	1.34	1.34	1.34
Symmetry	C5	C6	C1	C1
In. nr of particles	16660	11780	11780	
Final nr. of particles	11780	8076	5974	
Initial model	C5 map of virion capsid low-pass filtered to 30A	*de novo*	*de novo*	
Map resolution	5.1	4.2	7.3	
FSC treshold	0.143	0.143	0.143	
**Database entry**				
EMDB	EMD-14490	EMD-14495	EMD-14491	EMD-14920
PDB		7Z4F		
**Refinement**				
Atoms (except hydrogens)		25733		
Residues		3600		
B-factor		218		
FSC model 0.5 (masked)		8.34		
**RMSD**				
Bond length [A]		0.006		
Bond angles [°]		0.828		
**Validation**				
MolProbity score		2.07		
ClashScore		11.5		
C-beta outliers [%]		0		
Poor rotamers [%]		0		
**Ramachandran plot**				
outliers [%]		0.62		
favoured [%]		91.83		

Supplementary Table 3. **Cryo-EM structure quality indicators of SU10 genome release intermediate.**

Uncropped scan of SDS PAGE gel of proteins from virions and genome release intermediates of bacteriophage SU10. Source file for the preparation of Supplementary Fig. 4C.

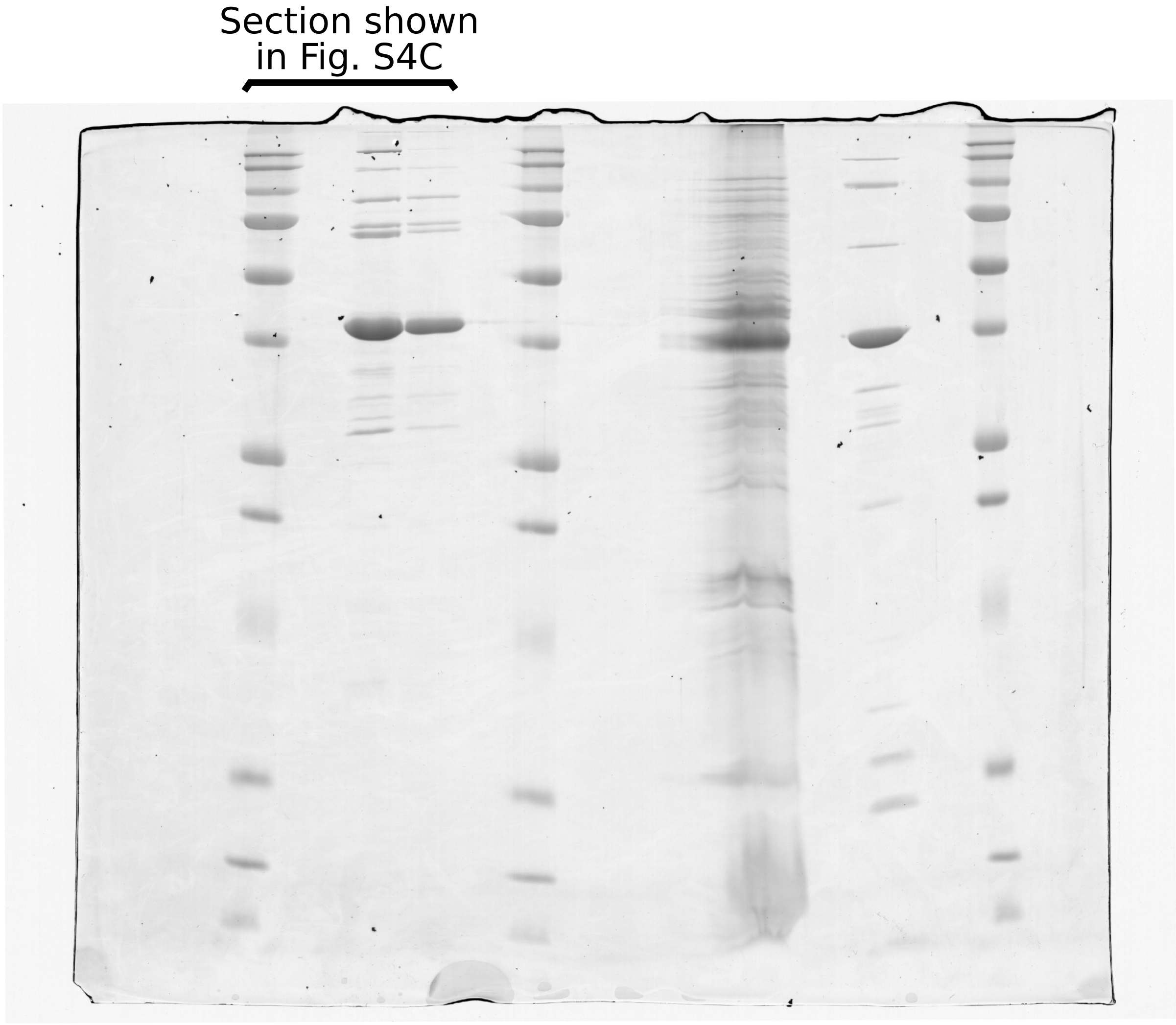

Supplement: Supplementary file 1 — Supplementary Information [file 41467_2022_33305_MOESM1_ESM.pdf]
