# Supplementary material for: Tail proteins of phage SU10 reorganize into the nozzle for genome delivery
Source: Nat Commun. 2022 Sep 24;13:5622. doi: 10.1038/s41467-022-33305-w (PMC9509320; doi:10.1038/s41467-022-33305-w)
Supplement: Supplementary file 3 — Description of Additional Supplementary Files [file 41467_2022_33305_MOESM3_ESM.pdf]

Description of Additional Supplementary Files

Supplementary movie 1. Conformational changes required for formation of nozzle from nozzle proteins and short tail fibers. The conformational changes required for the formation of the SU10 nozzle are shown for one-sixth of the tail. Cartoon representation of nozzle protein with platform domain shown in orange, beta-propeller domain in blue, short tail fiber dock in magenta, and four nozzle domains in red. The short tail fiber is shown in green.

Supplementary movie 2. Cryoelectron tomograph of SU10 infected E. coli cell. Segment of infected E. coli cell 45 minutes post-infection contains SU10 phages at various stages of the infection cycle. Virions outside the cell are shown in turquoise, genome release intermediates with formed nozzles in dark blue, empty particles in cyan, and progeny virions inside the cell in red. Inner and outer cell membranes are highlighted in dark orange and orange respectively, vesicles in light orange, ribosomes in light blue, the chemoreceptor array in green, and flagellum in violet.
