# Supplementary material for: Tail proteins of phage SU10 reorganize into the nozzle for genome delivery
Source: Nat Commun. 2022 Sep 24;13:5622. doi: 10.1038/s41467-022-33305-w (PMC9509320; doi:10.1038/s41467-022-33305-w)
Supplement: Supplementary file 6 — Reporting Summary [file 41467_2022_33305_MOESM6_ESM.pdf]

nature portfolio

Corresponding author(s): Pavel Plevka

Last updated by author(s): Aug 19, 2022

Reporting Summary

Nature Portfolio wishes to improve the reproducibility of the work that we publish. This form provides structure for consistency and transparency in reporting. For further information on Nature Portfolio policies, see our Editorial Policies and the Editorial Policy Checklist.

Statistics

For all statistical analyses, confirm that the following items are present in the figure legend, table legend, main text, or Methods section.

n/a	Confirmed	
[x]	[ ]	The exact sample size (*n*) for each experimental group/condition, given as a discrete number and unit of measurement
[x]	[ ]	A statement on whether measurements were taken from distinct samples or whether the same sample was measured repeatedly
[x]	[ ]	The statistical test(s) used AND whether they are one- or two-sided<br>*Only common tests should be described solely by name; describe more complex techniques in the Methods section.*
[x]	[ ]	A description of all covariates tested
[x]	[ ]	A description of any assumptions or corrections, such as tests of normality and adjustment for multiple comparisons
[x]	[ ]	A full description of the statistical parameters including central tendency (e.g. means) or other basic estimates (e.g. regression coefficient) AND variation (e.g. standard deviation) or associated estimates of uncertainty (e.g. confidence intervals)
[x]	[ ]	For null hypothesis testing, the test statistic (e.g. *F*, *t*, *r*) with confidence intervals, effect sizes, degrees of freedom and *P* value noted<br>*Give P values as exact values whenever suitable.*
[x]	[ ]	For Bayesian analysis, information on the choice of priors and Markov chain Monte Carlo settings
[x]	[ ]	For hierarchical and complex designs, identification of the appropriate level for tests and full reporting of outcomes
[x]	[ ]	Estimates of effect sizes (e.g. Cohen's *d*, Pearson's *r*), indicating how they were calculated

Our web collection on statistics for biologists contains articles on many of the points above.

Software and code

Policy information about availability of computer code

Data collection	EPU v1.12 and SerialEM 3.8.18 software packages were used to control the electron microscopy data collection.
Data analysis	Electron microscopy data were motion-corrected globally and locally (5 × 5 patches) using the software MotionCor2/1.2.6. Defocus values were estimated from aligned non-dose-weighted micrographs using the program Gctf 1.06. Single particle reconstruction was performed using the programs CrYOLO 1.5.4 from the package SPHIRE, Relion 3.1, and Xmipp 3.20.07. Software packages eTomo, IMOD, and PEET version 4.7.15 were used to analyze tomography data. Protein structures were predicted using AlphaFold multimer 2.1.2.<br>Scripts for positioning high-resolution structures into the tomograms are available from https://github.com/fuzikt/tomostarpy.

For manuscripts utilizing custom algorithms or software that are central to the research but not yet described in published literature, software must be made available to editors and reviewers. We strongly encourage code deposition in a community repository (e.g. GitHub). See the Nature Portfolio guidelines for submitting code & software for further information.

Data

Policy information about availability of data

All manuscripts must include a data availability statement. This statement should provide the following information, where applicable:

- Accession codes, unique identifiers, or web links for publicly available datasets
- A description of any restrictions on data availability
- For clinical datasets or third party data, please ensure that the statement adheres to our policy

The cryo-EM reconstructions and corresponding PDB structures were deposited under the following EMDB and PDB codes: Structures of SU10 virion: capsid with fivefold symmetry EMD-14488 and PDB-7Z49, asymmetric capsid reconstruction EMD-14492 and PDB-7Z4B, capsid top EMD-14485 and PDB-7Z46, capsid center EMD-14484 and PDB-7Z45, capsid bottom EMD-14487 and PDB-7Z48, assymmetric reconstruction of capsid bottom and tail EMD-14489 and PDB-7Z4A, neck with

twelvefold symmetry EMD-14483 and PDB-7Z44, tail with sixfold symmetry EMD-14486 and PDB-7Z47, tail needle with threefold symmetry EMD-14909, and composite map of the whole virion EMD-14977. Structures of genome release intermediate: capsid with fivefold symmetry EMD-14490, tail with sixfold symmetry EMD-14495 and PDB-7Z4F, capsid top EMD-14491, and composite map of the whole genome release intermediate EMD-14920. Source data for Supplementary Tables 1-3 are provided with this paper.

Field-specific reporting

Please select the one below that is the best fit for your research. If you are not sure, read the appropriate sections before making your selection.

- [x] Life sciences
- [ ] Behavioural & social sciences
- [ ] Ecological, evolutionary & environmental sciences

For a reference copy of the document with all sections, see nature.com/documents/nr-reporting-summary-flat.pdf

Life sciences study design

All studies must disclose on these points even when the disclosure is negative.

Sample size	The sizes of cryo-EM datasets were determined by the time available for data collection. The sample sizes were sufficient to determine the macromolecular structures presented in our manuscript.
Data exclusions	Standard approaches for image analyses were employed during cryo-EM data reconstruction procedures. Images of individual particles were rejected from the reconstructions using 2D and 3D classification approaches with pre-defined criteria.
Replication	Multiple cryo-EM datasets of SU10 virions and genome release intermediates were recorded.
Randomization	Particle images were randomly sorted into two sub-groups for independent reconstructions using automated procedure implemented in the cryo-EM reconstruction pipeline.
Blinding	Our conclusions are not based on interpreting individual images, instead the reconstructions were calculated from datasets including thousands of particle images. Therefore, blinding is not relevant for interpreting our results.

Reporting for specific materials, systems and methods

We require information from authors about some types of materials, experimental systems and methods used in many studies. Here, indicate whether each material, system or method listed is relevant to your study. If you are not sure if a list item applies to your research, read the appropriate section before selecting a response.

Materials & experimental systems

n/a	Involved in the study	
x		Antibodies
x		Eukaryotic cell lines
x		Palaeontology and archaeology
x		Animals and other organisms
x		Human research participants
x		Clinical data
x		Dual use research of concern

Methods

n/a	Involved in the study	
x		ChIP-seq
x		Flow cytometry
x		MRI-based neuroimaging
